# Prevalence of Invasive Bacterial Infection Among Febrile Infants Aged 61 to 90 Days

**DOI:** 10.1001/jamanetworkopen.2025.7710

**Published:** 2025-04-28

**Authors:** Etimbuk Umana, Thomas Waterfield

**Affiliations:** 1 Wellcome-Wolfson Institute for Experimental Medicine, Queen’s University Belfast, Belfast, United Kingdom; 2 Emergency Department, Royal Belfast Hospital for Sick Children, Belfast, United Kingdom

## Abstract

This cohort study evaluated the risk of invasive bacterial infection among infants aged 61 to 90 days and whether clinical appearance was associated with invasive bacterial infection risk in this population.

## Introduction

Febrile infants younger than 90 days are at higher risk of invasive bacterial infection (IBI), including bacteremia and meningitis, compared with older infants and children.^1,2^ Clinical appearance is also associated with IBI risk.^2,3^ This increased risk is reflected in international guidelines recommending that infants younger than 61 days should undergo a minimum of a clinical assessment, infection biomarker testing, and urinalysis.^4^ In the UK and Europe this approach extends to include infants younger than 91 days due to an IBI risk higher than 1% in those aged 61 to 90 days.^2,5^ This contrasts with US guidance that classifies infants older than 60 days as lower risk, which does not require routine biomarker testing and urinalysis.^4^ This study evaluated IBI risk among infants aged 61 to 90 days and whether clinical appearance was associated with IBI risk in this population.

## Methods

This cohort study was a secondary analysis of the FIDO (Febrile Infants Diagnostic assessment and Outcome) study.^3^ The study period was from July 2022 to August 2023. Ethics approval for the FIDO study extended to the present study, and written informed consent was obtained from participants. We followed the STROBE reporting guideline. Eligible participants were infants aged 90 days or younger with a temperature 38 °C or more in the emergency department or assessment unit and within 24 hours before presentation. The study procedure has been previously described.^3^ Infants were considered clinically unwell in appearance based on a clinician’s global assessment and/or vital signs outside the reference range^3^ (eMethods in Supplement 1). IBI was defined as a bacterial pathogen identified in either blood or cerebrospinal fluid by culture or molecular testing. IBI rates were reported in the following age groups: aged 28 days or less, 29 to 60 days, 61 to 90 days, and age in weeks. Subgroup analysis was conducted for clinical appearance (well vs unwell). Results were reported using descriptive statics. Categorical variables were analyzed using a χ^2^ or Fisher exact test. Two-sided *P* < .05 indicated statistical significance. Statistical analysis was performed from January to February 2025 using SPSS, version 23 (IBM).

## Results

Among 1821 febrile infants included in the study, 1108 (61%) were males. The median age was 46 (IQR, 30–64) days, 434 (23.8%) were aged 28 days or younger, 835 (45.9%) were 29 to 60 days, and 552 (30.3%) were 61 to 90 days. Regarding clinical appearance, 763 (41.9%) appeared clinically well, while 1058 (58.1%) appeared clinically unwell. A total of 67 infants (3.7%) had IBI, including 62 (3.4%) with bacteremia and 9 (0.5%) with bacterial meningitis. IBI rates in febrile infants aged 28 days or younger, 29 to 60 days, and 61 to 90 days were 6.7%, 2.8%, and 2.7% respectively. There was no difference in IBI rates in terms of clinical appearance for the 28 days or younger and 29 to 60 days groups (Table). For the group aged 61 to 90 days, there was a difference in IBI rates between infants with well (0.4%) and unwell (4.8%) clinical appearance (*P* < .001). There were no cases of meningitis in the clinically well group aged 61 to 90 days. The IBI rate decreased per week of age from 8.8% (1-7 days) to 3.8% (83-90 days) (Figure), with higher rates of IBI among infants with clinically unwell appearance in all age bands.

**Table.** Invasive Bacterial Infection (IBI) Rates in Different Age Groups

Age band and IBI status	Well clinical appearance No. (%) (n = 763)	IBI rate, % (95% CI)	Unwell clinical appearance, No. (%) (n = 1058)	IBI rate, % (95% CI)	*P* value
≤28 d					
Total	178 (23.3)	4.5 (2.0-8.7)	256 (24.2)	8.2 (5.2-12.3)	.13
With IBI	8 (1.0)		21 (2.0)		
Without IBI	170 (23.3)		235 (22.2)		
29-60 d					
Total	324 (42.5)	2.2 (0.9-4.4)	511 (48.3)	3.1 (1.8-5.0)	.40
With IBI	7 (0.9)		16 (1.5)		
Without IBI	317 (41.5)		495 (46.8)		
61-90 d					
Total	261 (34.2)	0.4 (0.0-2.1)	291 (27.5)	4.8 (2.7-7.9)	.001
With IBI	1 (0.1)		14 (1.3)		
Without IBI	260 (34.1)		277 (26.2)		

**Figure.**
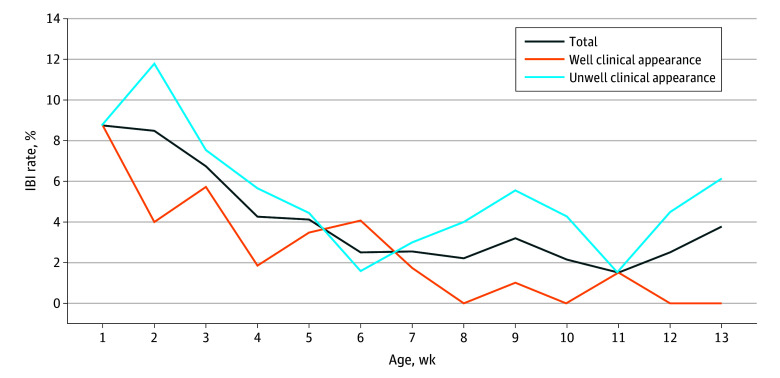
Invasive Bacterial Infection Rates by Age in Weeks and Appearance Prevalence of invasive bacterial infection (IBI) by age in weeks (0-90 days) is shown overall and by well and unwell clinical appearance.

## Discussion

This study found that in the UK and Ireland, IBI rates decrease with age as reported in other international cohort studies.^2,6^ The IBI rate among infants aged 61 to 90 days was 2.7%, which was similar to the 2.8% rate in infants aged 29 to 60 days, supporting a similar assessment method for the 2 groups. IBI risk was lower in infants aged 61 to 90 days with a clinically well appearance. Hence, a tailored approach should be adopted, incorporating shared decision-making. Study limitations include data specific to UK and Ireland and potential underestimation of IBI rates due to medical record review methods.

## Notes

Supplement 1.
eMethods.

Supplement 2.
Data Sharing Statement
