# Supplementary material for: Prevalence of Invasive Bacterial Infection Among Febrile Infants Aged 61 to 90 Days
Source: JAMA Netw Open. 2025 Apr 28;8(4):e257710. doi: 10.1001/jamanetworkopen.2025.7710 (PMC12038498; doi:10.1001/jamanetworkopen.2025.7710)
Supplement: Supplement 1. — eMethods. [file jamanetwopen-e257710-s001.pdf]

Supplemental Online Content

Umana E, Waterfield T. Prevalence of invasive bacterial infection among febrile infants aged 61 to 90 days. *JAMA Netw Open*. 2025;8(4):e257710. doi:10.1001/jamanetworkopen.2025.7710

eMethods.

This supplemental material has been provided by the authors to give readers additional information about their work.

© 2025 Umana E. et al. *JAMA Network Open*.

eMethods.

The FIDO study was conducted across 35 sites within the paediatric emergency research network in the UK and Ireland (PERUKI). Eligible patients were consecutively recruited. An infant was deemed unwell if they showed abnormal global assessment or vital signs at presentation. Clinicians assessed infants based on clinical judgment, categorizing them as having normal or abnormal global status. Vital signs were marked abnormal if heart rate, respiratory rate, or capillary refill time were outside the Advanced Paediatric Life Support reference range. Participants who did not have culture/qPCR testing were assumed not to have IBI if they were not subsequently found to have been diagnosed with IBI within seven days of discharge on chart review

© 2025 Umana E. et al. *JAMA Network Open*.
