# Supplementary material for: Prevalence of Invasive Bacterial Infection Among Febrile Infants Aged 61 to 90 Days
Source: JAMA Netw Open. 2025 Apr 28;8(4):e257710. doi: 10.1001/jamanetworkopen.2025.7710 (PMC12038498; doi:10.1001/jamanetworkopen.2025.7710)
Supplement: Supplement 2. — Data Sharing Statement [file jamanetwopen-e257710-s002.pdf]

Data Sharing Statement

Umana. Prevalence of Invasive Bacterial Infection Among Febrile Infants Aged 61 to 90 Days. *JAMA Netw Open*. Published April 28, 2025. doi:10.1001/jamanetworkopen.2025.7710

Data
Data available: Yes
Data types: Deidentified participant data
How to access data: Available on PURE repository at Queens University Belfast. Available on reasonable request
When available: beginning date: 12-19-2025

Supporting Documents
Document types: None

Additional Information
Who can access the data: Researchesrs
Types of analyses: secondary analysis
Mechanisms of data availability: With investigator support
